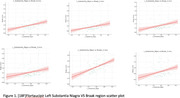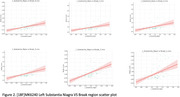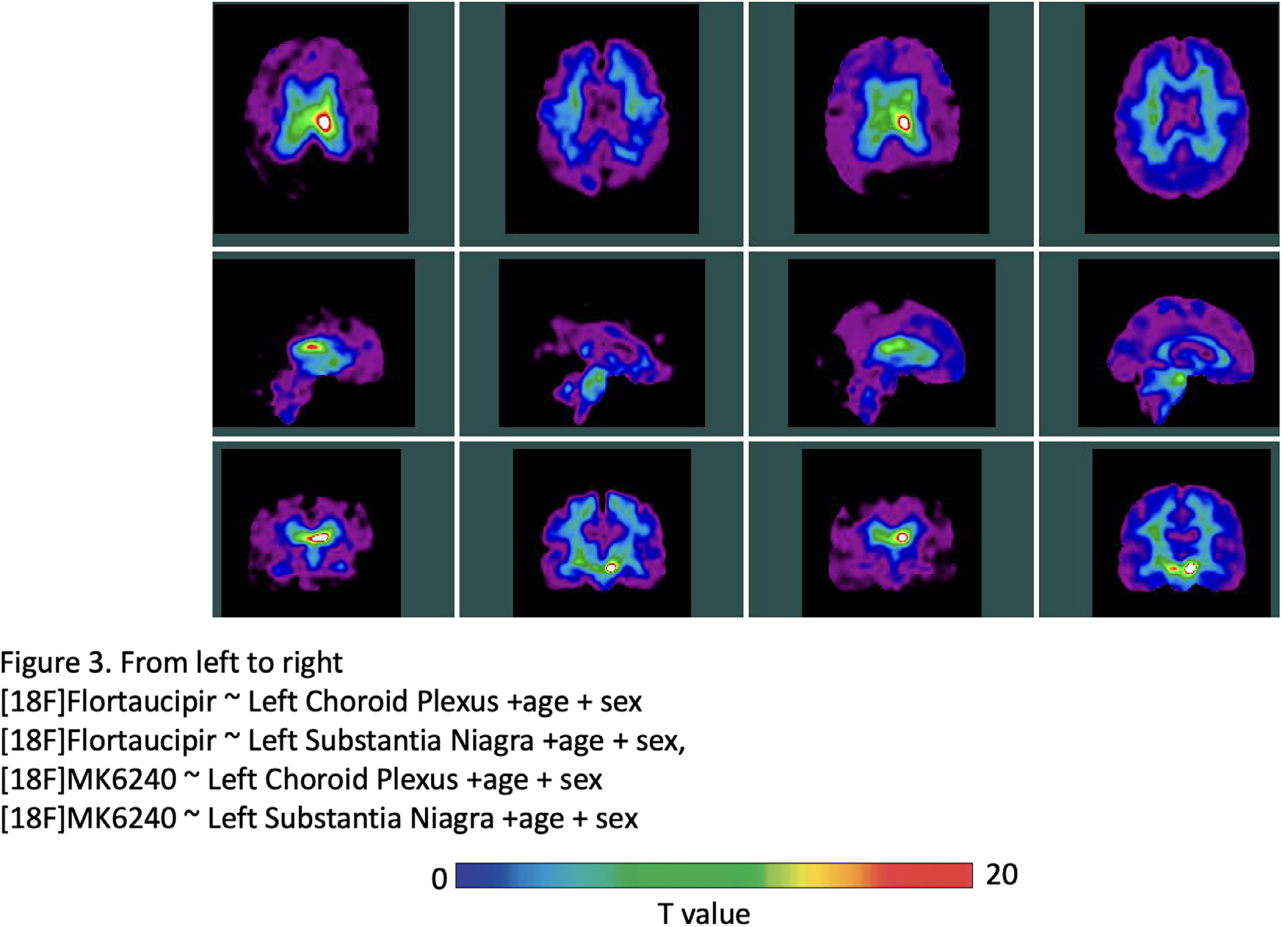# Patterns of Association of Tau Across Cortical Regions Differ Between [^18^F]MK6240 and [^18^F]Flortaucipir

**DOI:** 10.1002/alz.093565

**Published:** 2025-01-09

**Authors:** Seyyed Ali Hosseini, Nesrine Rahmouni, Joseph Therriault, Stijn Servaes, Cécile Tissot, Etienne Aumont, Jaime Fernandez Arias, Arthur C. Macedo, Yi‐Ting Wang, Jenna Stevenson, Firoza Z Lussier, Tevy Chan, Yansheng Zheng, Lydia Trudel, Serge Gauthier, Suzanne L. Baker, Tharick A. Pascoal, Pedro Rosa‐Neto

**Affiliations:** ^1^ Translational Neuroimaging Laboratory, The McGill University Research Centre for Studies in Aging, Montréal, QC Canada; ^2^ Montreal Neurological Institute, Montreal, QC Canada; ^3^ Université du Québec à Montréal, Montréal, QC Canada; ^4^ University of Pittsburgh, Pittsburgh, PA USA; ^5^ Douglas Mental Health University Institute, Montréal, QC Canada; ^6^ Lawrence Berkeley National Laboratory, Berkeley, CA USA

## Abstract

**Background:**

This study aims to investigate the differential patterns of association in tau protein imaging across cortical regions using two distinct Tau imaging agents: [18F]MK6240 and [18F]Flortaucipir. The underlying hypothesis posits that variations in the properties of these tracers, such as affinity and off‐target effects, influence the observed patterns of association in neuroimaging.

**Method:**

To test this hypothesis, a comprehensive study was conducted involving 104 subjects part of the HEAD study at McGill University: 53 cognitively normal (CN), 19 with mild cognitive impairment (MCI), 9 with Alzheimer's Disease (AD) and 23 non‐AD. A key methodological aspect of the study was the selection of seed cortical regions based on the highest standardized uptake value ratios (SUVR) obtained in population average images. These regions included the trans‐entorhinal area, parietal, posterior cingulate cortices, and two off‐target binding regions: the choroid plexus and substantial nigra. This approach allowed for a nuanced analysis of tracer associations in both target and off‐target regions.

**Result:**

The results revealed notable differences between the two tracers. Specifically, [18F]MK6240 exhibited higher associations between cortical regions, with similar slopes in these associations across different areas. In contrast, the associations between the off‐target binding regions (Choroid Plexus and Substantia Nigra) and the established Braak regions were found to be non‐significant. This finding is particularly revealing, as it underscores the differential binding affinity and specificity of the two tracers.

**Conclusion:**

In conclusion, the study substantiates the initial hypothesis by demonstrating that distinct tau tracers exhibit unique patterns of cortical associations, which can be attributed to their specific properties. This insight has significant implications for the interpretation of tau imaging in neurodegenerative diseases, particularly in AD. It highlights the necessity of considering tracer‐specific characteristics when analyzing neuroimaging data and suggests that a one‐size‐fits‐all approach may not be appropriate in the nuanced field of neuroimaging. The findings of this study pave the way for more refined and accurate diagnostic practices in neurology, particularly in the context of neurodegenerative diseases where tau protein plays a pivotal role.